# Current Controversies on the Use of Magnetic Resonance Imaging in the Management of Breast Cancer

**DOI:** 10.4021/wjon309w

**Published:** 2011-06-08

**Authors:** Edibaldo Silva

**Affiliations:** Department of Surgery, Division of Surgical Oncology, University of Nebraska Medical Center, 984030 Nebraska Medical Center, Omaha, NE 68198, USA. Email: esilva@unmc.edu

**Keywords:** Breast MRI, Sporadic breast cancer, Breast conserving surgery, Contralateral prophylactic mastectomy

## Abstract

The use of magnetic resonance imaging in the management of unselected female populations with early stage breast cancer has increased markedly over the past decade. Parallel to this trend many have observed an increased use of mastectomy over breast conservation due to concerns raised by the use of MRI. Similar concerns have led to the use of contra-lateral prophylactic mastectomy in patient populations not tested for potential genetic predisposition. These trends are difficult to understand as they divert from well established clinical paradigms which have been the result of widely accepted clinical research trials with more than three decades of clinical follow up. These trials have asserted that breast conserving surgery remains the accepted approach over mastectomy for the care of the patient with sporadic early stage breast cancer.

## Introduction

Advances in diagnostic imaging have usually led to improvement in patient management in medicine. Recently, advances in breast diagnostic imaging with MRI in unselected patient populations have uncovered a number of pitfalls when evaluated in the context of the well established clinical practice paradigms derived from pivotal clinical trials. Recognition of these pitfalls should lead to clinical trials to resolve the more controversial issues and result in practice algorithms to support the use of breast MRI in targeted patient populations.

Diagnostic imaging with mammography has resulted in an increase in the number of women whose cancer is less than 2 cm in size. Decrease in stage at presentation of these smaller tumors has led to a survival benefit beyond that expected by simple stage migration alone for patients with screen detected cancer versus those whose cancers are detected by clinical exam [1]. Mortality decreases from breast cancer resulting from mammographic screening are estimated to be 46% and vary by the age of the cohort examined [2]. Still, mammography misses approximately 20% of all breast cancer particularly in young women with dense breast parenchyma [3].

## MRI Versus Mammographic Screening of Unselected Populations

Advances in breast imaging with MRI have led to increased sensitivity of breast cancer detection although at an increased cost. This increased sensitivity is plagued by significant variation in specificity with most studies showing the specificity of MRI to be lower than that of screening mammography [4]. Comparison of the specificity and sensitivity of MRI versus mammography in breast cancer screening has been studied primarily in populations of women at high risk for developing breast cancer such as BRCA gene carriers. Even in these groups where young age compromises the reliability of mammography, MRI resulted in additional recall examinations in 10.7% of women compared to 3.9% for mammography [5]. In turn, these additional exams led to a 3-fold increase in recommendations for biopsy (3.1% vs. 1.3%). The increased cost of MRI with its attendant increase in repeat exams and biopsies seen in high risk populations is likely to forestall its application to the routine screening of all women where the putative marginal advantage of MRI’s sensitivity over digital mammography in the 50 year old or older group may be slim.

## MRI and Breast Conserving Surgery

### The potential ipsilateral second primary

The role of MRI in the management of the newly diagnosed patient with breast cancer represents the area of controversy where the accumulated evidence of the past 30 years provides significant insight into the pitfalls associated with routine MRI use. It is here where the MRI interpretation has the most significant impact and potential deleterious effect on the care of the patient with breast cancer. To understand these pitfalls we must review some of the remarkable progress in our understanding of the natural history and treatment of breast cancer made in the past 40 years.

In 2002, reports by Fisher [6] and Veronesi [7] affirmed the long term validity of breast conserving surgery (BCS) for the management of early stage breast cancer (ESBC). Years earlier in 1991, a NCI Consensus Conference endorsed BCS, defined as margin clear lumpectomy with post-operative whole breast irradiation, over mastectomy as the preferred treatment of ESBC. This recommendation has been strengthened by accumulating biological and clinical evidence of the natural history of this disease in patients with a single breast cancer documented by mammographic evaluation. When treated with BCS or mastectomy local recurrence rates are the same, 5 - 10% in patients with (ESBC) [8]. As discussed above, the success of BCS stems from the increased use of screening mammography resulting in the early diagnosis of smaller breast cancer.

Traditionally, BCS has been contraindicated in patients with multicentric disease (tumors in other quadrants of the breast) as documented by mammographic exam at the time of diagnosis. The increasing use of MRI in patients with a known breast cancer has led to the identification of multiple lesions in patients who heretofore had only a single primary tumor identified by physical exam or mammography. The increased sensitivity of MRI in this setting leads to the identification of potential second cancers in the same breast of 13 - 30% of patients [9, 10]. Curiously, the presence of multifocal tumors in patients with even the smallest of breast cancers was noted by Holland in detailed serial section histologic exam of mastectomy specimens more than 20 years ago. In fact, Holland’s data suggested that as many as two-thirds of these patients had a second lesion within the same breast with most of them mapping within 4 cm of the known primary [11]. Holland’s data remains timely because it was used then as an admonition about the validity of BCS. The re-discovery of some of these lesions by MRI is now revisited as a reason for the conversion from BCS to mastectomy in patients with ESBC [12]. Paradoxically, analysis similar to that of Holland was recently reported by Japanese investigators supporting the use of accelerated partial breast irradiation (APBI) which spares the breast tissue outside of this 4 cm radius from any radiation in BCS. In this setting none of the multifocal lesions outside of the quadrant in question would have been subjected to radiation therapy and thus a higher local failure rate would have been expected [13]. Favorable four-year recurrence rates of 1% have been published for APBI with longer follow up continuing [14].

In a more remarkable study by Sardanelli [15], MRI was performed in women who had elected to undergo mastectomy for a single known cancer. Subsequent pathologic step section of the entire breast specimen using 5 mm sections in the manner of Holland then attempted to localize the patient’s primary cancer as well as any other suspected cancers detected by the pre-operative MRI. In 99 breast specimens, mammography and MRI missed 64 and 36 malignant tumors respectively 8 mm and 5 mm in median size. In addition, MRI failed to detect 19% of the malignant foci discovered by the pathologic evaluation of the breast. Although MRI was more sensitive than mammography, the predictive positive value (PPV) was low (only 70%), for both modalities. Therefore, even if patients where to be selected for BCS by MRI, nineteen percent would have had a second unknown primary cancer in the breast. This figure is clearly much higher than any reported local recurrence rate in patients treated with BCS and appropriate systemic therapy. Not surprisingly, a recent report shows that BCS patients selected on the basis of unifocal disease by MRI have identical local recurrence rate (3 - 4%) at 8 year follow up when compared to a similarly treated cohort undergoing BCS based on unifocal disease established by routine mammography only [16].

This compares very favorably to the recent update by the Early Breast Cancer Trialists (EBCT) which shows 10 year local failure rates of 8% for node negative women undergoing a more deforming mastectomy and 27% for node positive women similarly treated [17]. Thus, with increasingly smaller cancers disclosed by effective mammographic screening, the added weight of 25 years of clinical experience and the improvement in systemic therapies to date it is difficult to see how the rediscovery of well known subclinical tumors which are well controlled by contemporary radiotherapy and systemic therapy should discourage physicians and patients alike from BCS. The conversion of BCS to mastectomy resulting from indiscriminate use of breast MRI is shown by the Mayo Clinic report in which preoperative MRI raised the odds of undergoing mastectomy by 60% and having surgery in 2006 vs. 2003 raised those odds by 70% [18]. Similar increases in mastectomy rates (19% to 27%) were just reported by Bleicher in patients undergoing preoperative MRI [19]. In Berg’s report [10], 12% of women chose an unneeded mastectomy without any biopsies to exclude malignancy of the questionable second ipsilateral lesions detected by MRI and which were determined at mastectomy NOT to be malignant. Thus, the adoption of MRI without any prospective data on its benefit in the management of ESBC undermines the long prospective and data driven track efficacy record of BCS compared to mastectomy. Indiscriminate use of breast MRI threatens to reverse breast conservation trends to the detriment of women in the USA.

### Margin clearance at lumpectomy

Among women who wish to undergo BCS, margin positive rates of 55 - 68% have been reported [20]. Three large studies have shown that 50 - 62% of these women undergo a mastectomy as their next procedure to clear positive margins [21, 22, 23]. On average, in 50% of these women no residual cancer can be identified in the mastectomy specimen [21]. The preoperative use of MRI has been suggested could decrease these margins positive rates. However, a large randomized trial involving 1625 women compared preoperative mammography vs. MRI and their impact on this problem. Both approaches resulted in identical margin positive rates of 19% [24]. Similar findings were noted in a meta-analysis of data on MRI use in breast cancer staging [25]. In this study, 13.6% of women intent on BCS underwent a mastectomy or more cosmetically deforming excisions due to false positive findings on MRI. It is recommended that no change in surgical plans be made unless preoperative biopsy of MRI ancillary findings is performed first.

### Synchronous contralateral breast cancer risk

Lastly and more remarkable is the parallel trend of removal of the opposite unaffected breast in women with breast cancer. The introduction of MRI in breast diagnostics coupled with incomplete risk assessment by clinicians and understandably fearful patients has led to unnecessary preventive contra-lateral prophylactic mastectomy (CPM) in patients with ESBC and no genetic predisposition. Many breast cancer patients opt for contra-lateral prophylactic mastectomy based on a MRI finding without histologic proof of cancer. Invariably many who make this recommendation may not have the ability to perform MRI guided core biopsy of lesions not seen by mammography or ultrasonography. The report by Tuttle has shown an increase in contra-lateral mastectomy from 4.2% in 1998 to 11% in 2003. In this report of 152,755 patients from the Surveillance, Epidemiology and End Results (SEER) cancer registry, 8.3% were age 60 or older at the time of contra-lateral mastectomy [26]. Understandably, this figure was 25% among women age 18 - 49 undergoing a unilateral mastectomy for the treatment of their breast cancer. This trend, similar to that noted above by the Mayo Clinic [18] may have been accelerated by the increased utilization of MRI as Tuttle’s report also shows that the contra-lateral mastectomy rate increased between 2000 and 2003. It is concerning that the most common reason for CPM was physician’s advice regarding the risk of contra-lateral breast cancer. This negligible risk has been documented since the days of the radical mastectomy and demonstrates how inadequate informed consent can be very damaging. The risk of contra-lateral disease is well established as 3% at 5 years [27]. Furthermore, any risk of contra-lateral disease is decreased by 50% in patients using tomoxifen and 20% in those treated with chemotherapy [17]. Clearly, prophylactic removal of the unaffected breast can have no impact on the survival of a patient in whom the stage of her index cancer is the primary determinant of survival. Remarkably, even in high risk breast cancer patients undergoing prophylactic mastectomy, MRI missed three of four lesions (of these 3/4th were DCIS) noted at final pathology and added a great cost [28].

## Conclusion

Thus, it would appear that increased utilization of MRI by physicians not familiar with all of its limitations coupled with patient’s inordinate fear and incomplete risk assessment lead to very difficult conversations with patients at the time of informed consent. Many breast cancer specialists find that all this information is hard to convey at a single visit as it may require discussion about BCS, fear of radiation therapy, surveillance strategies, prevention strategies, and accurate genetic risk assessment. The pitfall here is that it is easier to recommend bilateral mastectomy than advice a bereft patient why it is perfectly reasonable to adhere to data driven practice patterns established over the last 30 years [29, 30]. Thus the current indiscriminate use of MRI in the screening and management of breast cancer is potentially associated with a significant negative impact to the breast cancer patient in the absence of any evidence that it improves surgical care or prognosis.
